# Supplementary material for: Quantification of epitope abundance reveals the effect of direct and cross-presentation on influenza CTL responses
Source: Nat Commun. 2019 Jun 28;10:2846. doi: 10.1038/s41467-019-10661-8 (PMC6599079; doi:10.1038/s41467-019-10661-8)
Supplement: Supplementary file 1 — Supplementary Information [file 41467_2019_10661_MOESM1_ESM.pdf]

SUPPLEMENTARY INFORMATION

Quantification of epitope abundance reveals the effect of direct and cross-presentation on influenza CTL responses

Wu et al

Supplementary Methods

Fitting the nonlinear machine-learning models

A support vector machine model (SVM), a random forest (RF) and a gradient boosted regression tree model (GBM) were fitted to the data. For comparison, we also included a LASSO model, which fits the linear model with a penalty for each variable, allowing variables to drop from the model and as such performs an alternative type of subset selection. Model tuning was performed by repeated cross-validation and performance optimization on the Mean Squared Error (MSE) for the test data. The final model performance as measured by average R^2 on the hold-out sets was 0.65/0.26/0.41/0.5 for the SVM, RF, GBM and LASSO models.

Thus, none of the models were able to improve on the performance of the linear model determined by subset selection. This is likely in large part due to the small sample size (N=20) which leads to overfitting of these more flexible algorithms, despite using a cross-validation approach that tries to minimize such overfits. Given the inferiority of these models, which suggest they are not suitable to describe the data, performing variable importance assessment is not meaningful. All computations were done using the mlr package in R. The support vector machine model was fit using the ksvm algorithm in the kernlab package in R, the boosted regression tree was fit using the gbm algorithm in the gbm package in R, the random forest was implemented using the ranger package, the LASSO algorithm from the glmnet package was used. All models were accessed through the mlr interface, which provided the cross-validation, tuning and performance assessment functionality. The full analysis code is provided as supplement (Supplementary Software 1).

Supplementary Figures

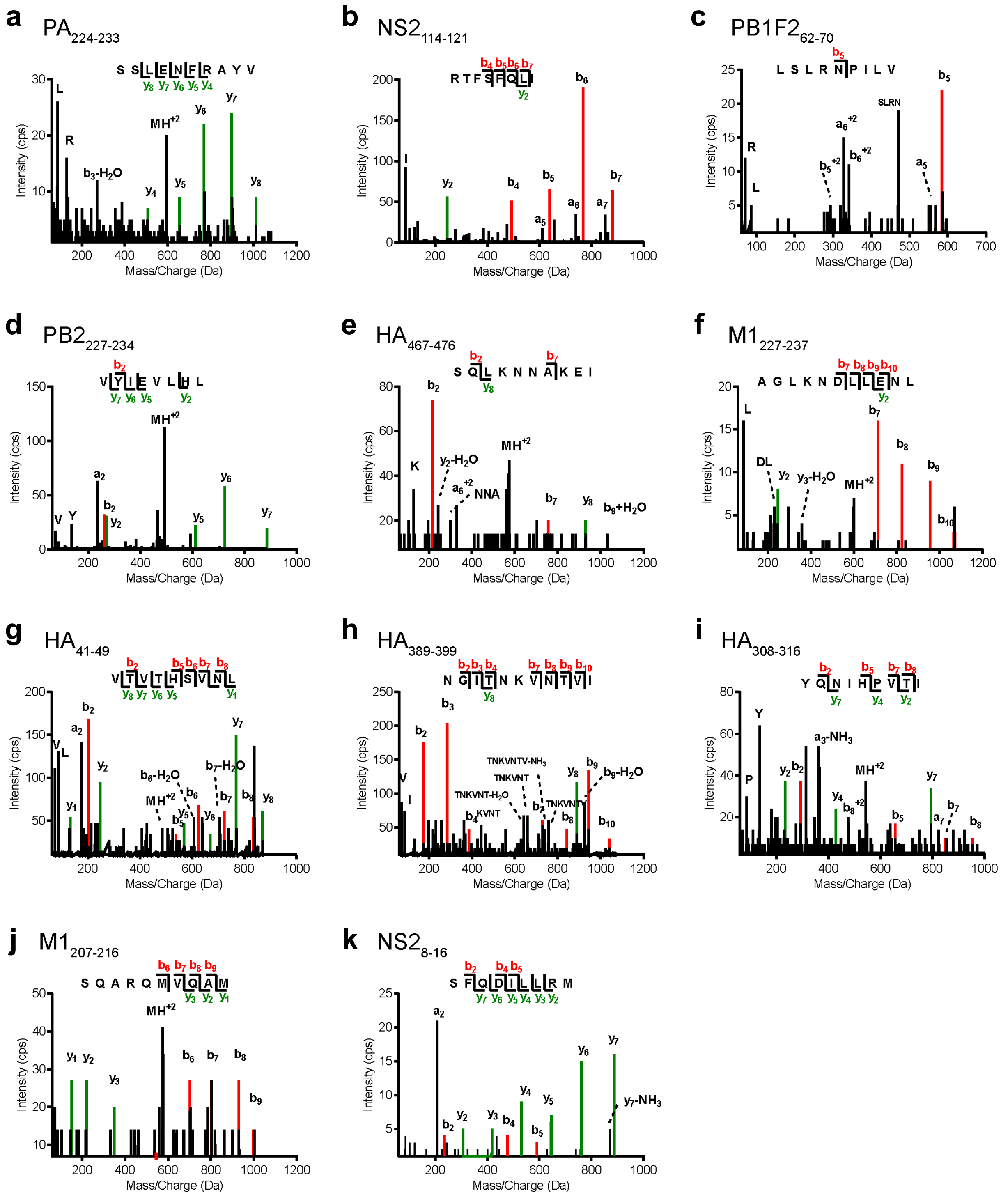

Supplementary Fig. 1. LC-MS/MS-based detection of H-2^b-restricted IAV-derived peptides. DC2.4 or LET-1 cells were infected (MOI 5) with IAV strain PR8 for 8 hrs, followed by cell lysis and immunoaffinity capture of H-2K^b or H-2D^b pMHCI. Following dissociation of complexes, peptides were fractionated by RP-HPLC and analysed by high-resolution LC-MS/MS. Shown are spectra assigned to 11 peptides derived from IAV polymerase (PA), non-structural protein (NS2),

alternate open reading frame of PB1 (PB1-F2), matrix protein 1 (M1) and haemagglutinin (HA). Panels F-K are peptides novel to this study.

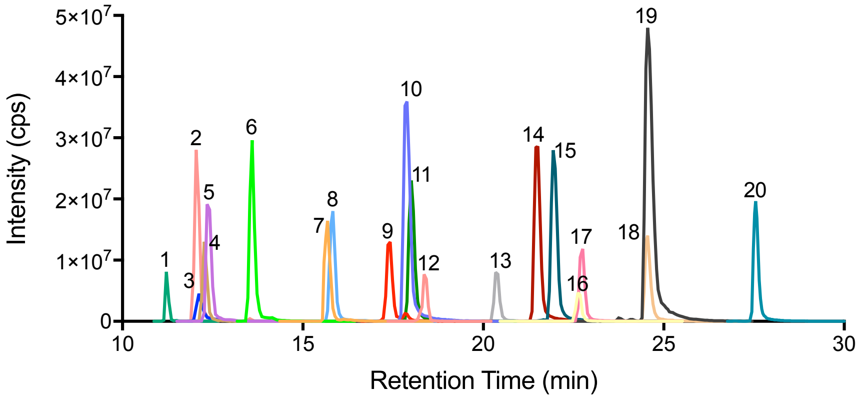

Supplementary Fig. 2. Multiplexed LC-MRM detection of 20 IAV peptides. A mixture of each isotopic synthetic peptide was analysed by non-scheduled LC-MRM. Shown is the sum intensity of all MRM transitions per peptide, with each peptide as follows: 1 – $PB1_{703\text{-}711}$; 2 – $HA_{402\text{-}409}$; 3 – $NP_{366\text{-}374}$; 4 – $NA_{181\text{-}190}$; 5 – $M1_{207\text{-}216}$; 6 – $HA_{41\text{-}49}$; 7 – $HA_{389\text{-}399}$; 8 – $HA_{308\text{-}316}$; 9 – $NA_{181\text{-}191}$; 10 – $M1_{128\text{-}135}$; 11 – $NP_{55\text{-}63}$; 12 – $HA_{304\text{-}311}$; 13 – $PA_{224\text{-}233}$; 14 – $NP_{36\text{-}43}$; 15 – $PB1F2_{62\text{-}70}$; 16 – $PB1_{653\text{-}660}$; 17 – $M1_{227\text{-}237}$; 18 – $NS2_{114\text{-}121}$; 19 – $PB2_{227\text{-}234}$; 20 – $NS2_{8\text{-}16}$.

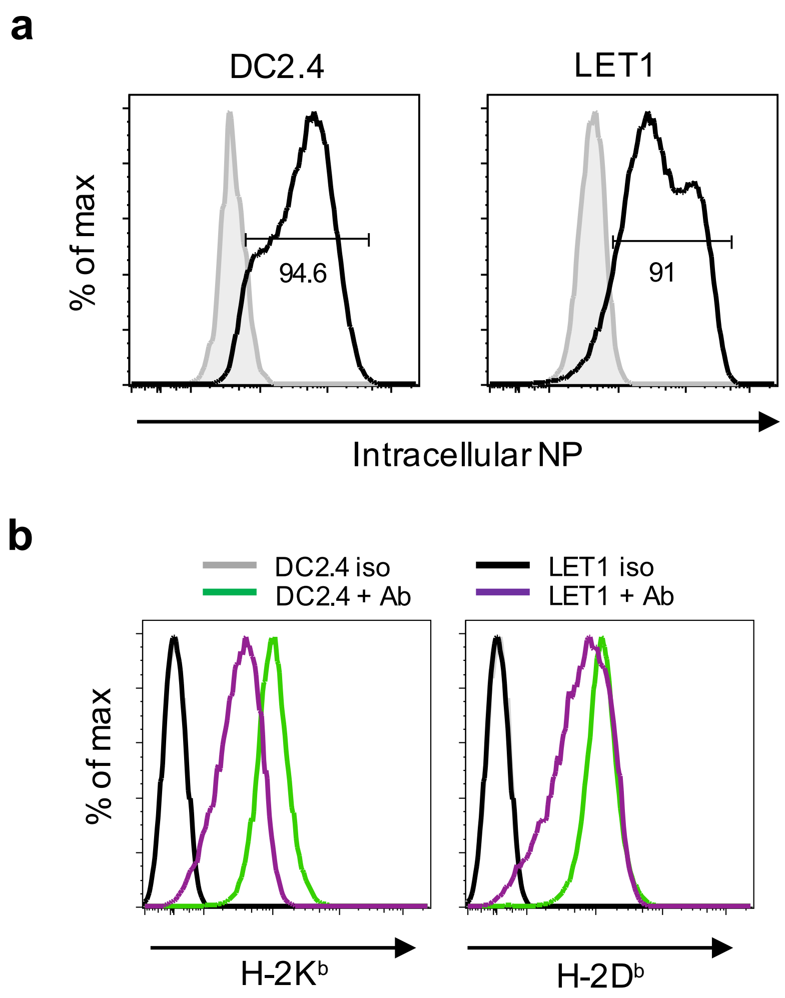

Supplementary Fig. 3. Infection rates and MHCI expression in DC2.4 and LET1 cells. (a) DC2.4 cells or LET1 cells ($1x10^8$) were incubated for 8h with or without PR8 IAV at MOI of 5. Cells were fixed and permeabilized for intracellular antibody staining of IAV nucleoprotein (NP). Expression was detected by flow cytometry. Shaded histogram represents mock treated cells, solid black histograms represent infected cells. (**b**) Levels of MHCI D^b and K^b molecules was determined by staining DC2.4 and LET1 cells with FITC conjugated antibodies to H-$2D^b$ and H-$2K^b$, and analysis by flow cytometry. Grey and black lines represent isotype control staining of DC2.4 and LET1 cells, respectively.

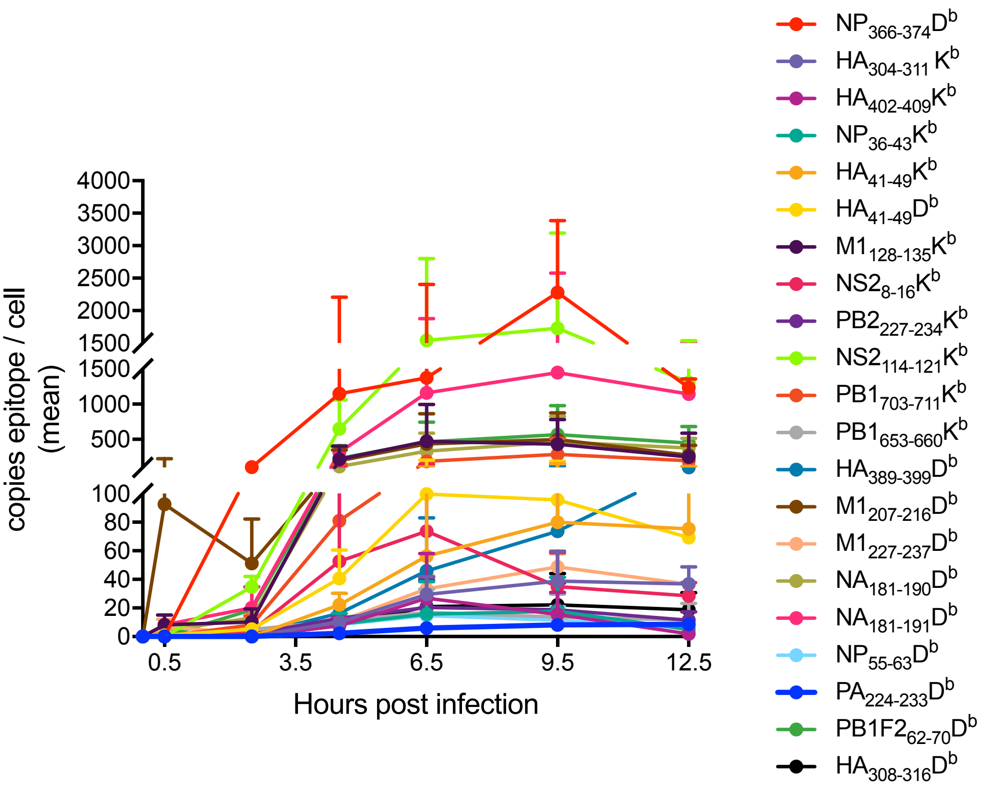

Supplementary Fig. 4. Kinetic analysis of peptide presentation. DC2.4 cells (1×10^8 per timepoint) were mock treated or infected with the PR8 strain of IAV at an MOI of 5 for 0, 0.5, 2.5, 6.5, 9.5 or 12.5 h, and epitopes were eluted from K^b and D^b MHCI molecules following immunocapture and analyzed by the LC-MRM workflow. Shown is the mean ± SEM number of peptide copies identified per cell. N = 3 independent timecourse experiments.

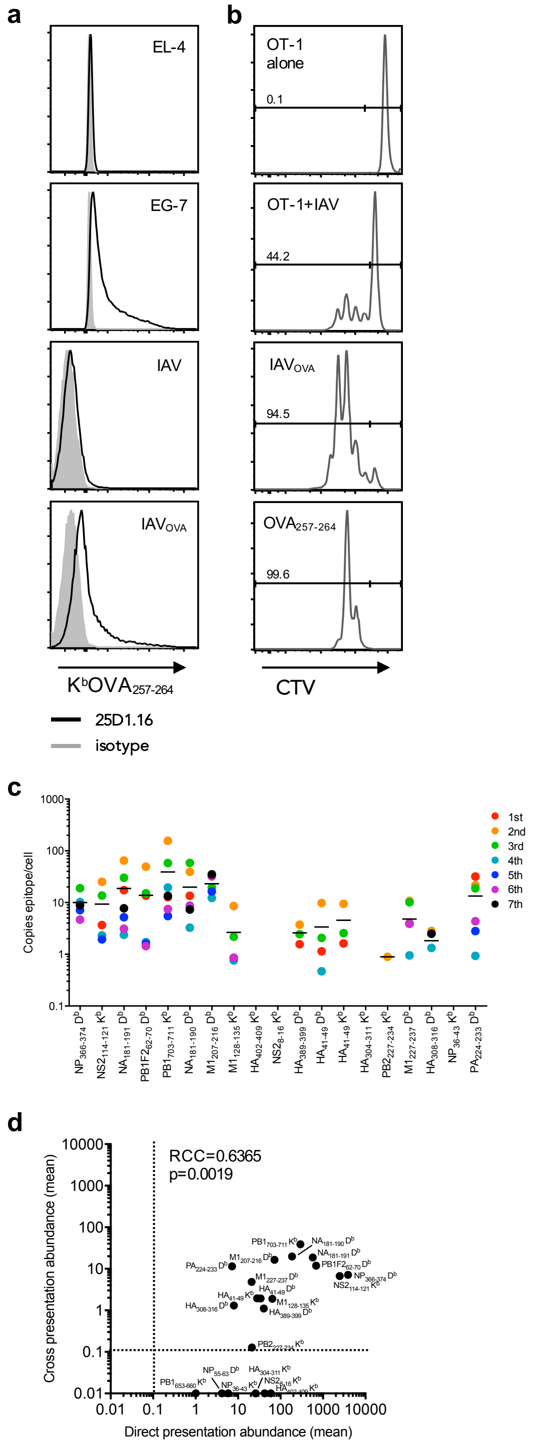

Supplementary Fig. 5. Validation of the in vitro cross-presentation system. A schematic of the *in vitro* cross-presentation system is shown in Figure 2. A549 cells (5x10^7) were infected for 10h with PR8 IAV or IAV expressing the ovalbumin peptide (257-264), SIINFEKL (IAV$_{OVA}$) at an MOI of 10. The A549 cells were then γ-irradiated (5000Gy) and co-incubated for 20 h with 5x10^7 Mutu DCs. (**a**) The Mutu DCs were then stained with the 25D1.16 antibody specific for the K^{b}OVA$_{257-264}$

complex (solid black line) or isotype control (grey shaded histogram). EL-4 cells (mouse thymoma cell line) or EG-7 cells (EL-4 cells stably transfected with DNA encoding chicken ovalbumin) were also stained as negative and positive controls, respectively. Staining was detected by flow cytometry. (**b**) Cross-presenting Mutu DCs were co-cultured with CTV-labelled OT-I TCR transgenic T cells for 48 h and OT-I cell division was determined by dilution of the CTV dye as detected by flow cytometry. (**c**) Absolute quantitation of peptide abundance following cross-presentation shown as peptide copies/cell. N = 7 independent infections. Colors indicate data obtained from the same experiment. (**d**) Linear regression analysis of epitope abundance via cross-presentation vs direct infection of DC2.4 cells, showing the Spearman's rank correlation coefficient (RCC) and associated p-value. Dashed black lines indicate the limit of detection.

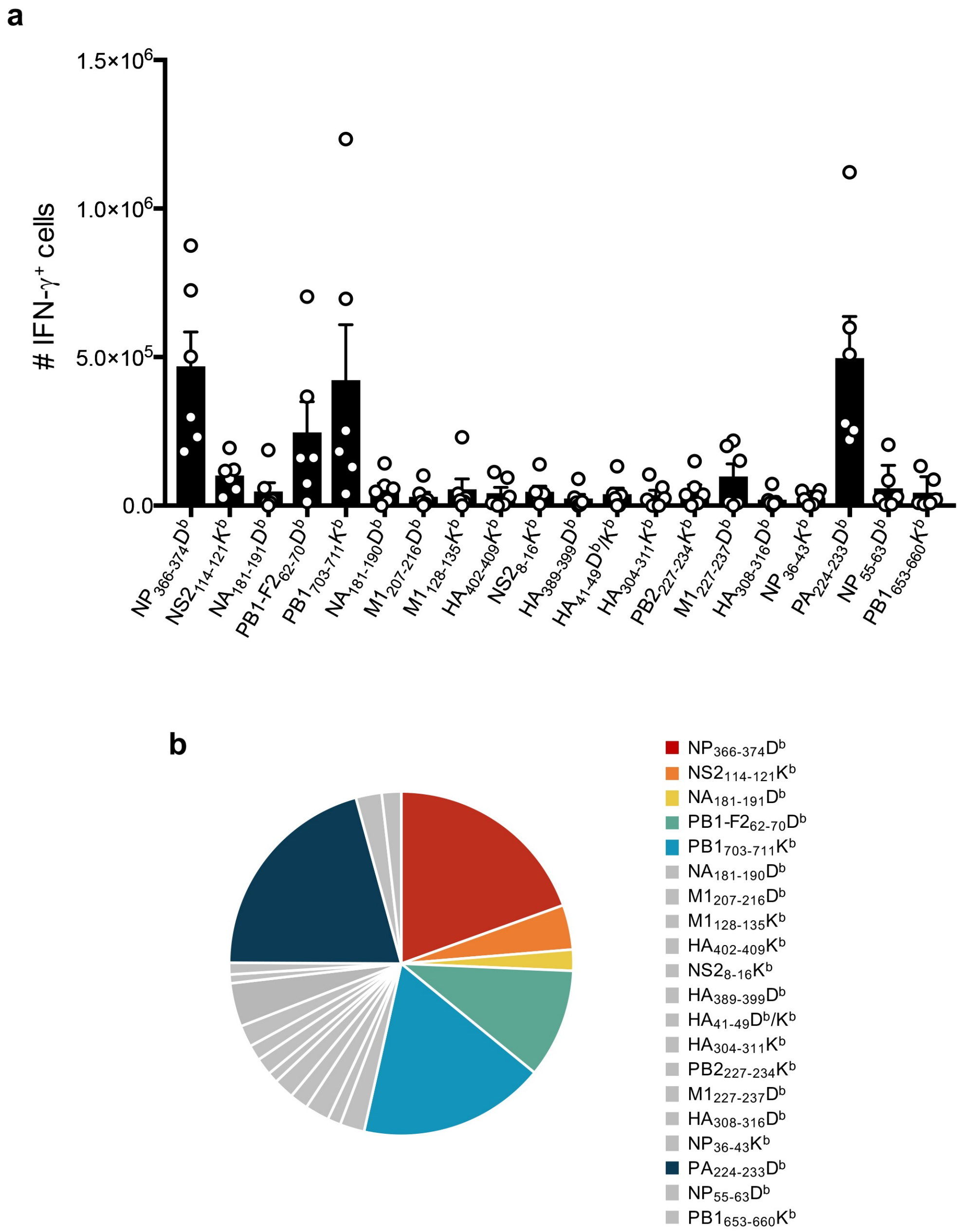

Supplementary Fig. 6. Analysis of primary CD8+ T cell responses to IAV-derived peptides identified by LC-MRM in the lungs. Naïve B6 mice were infected i.n. with 1000 pfu PR8 IAV, and bronchoalveolar lavage (BAL) harvested 10 days later. BAL cells, enriched for $CD8^+$ T cells, were stimulated for 5 hours in the presence or absence of 1 uM individual peptides, and analyzed by intracellular staining for the production of IFN-γ and TNF. (**a**) Mean number ± SEM of $CD8^+$ IFN-γ^+ cells responding to each peptide. N =11 mice from 2 independent experiments. (**b**) Pie chart showing the relative contribution of each detectable epitope specific $CD8^+$ T cell response to the total detectable response. Source data are provided as a Source Data File.

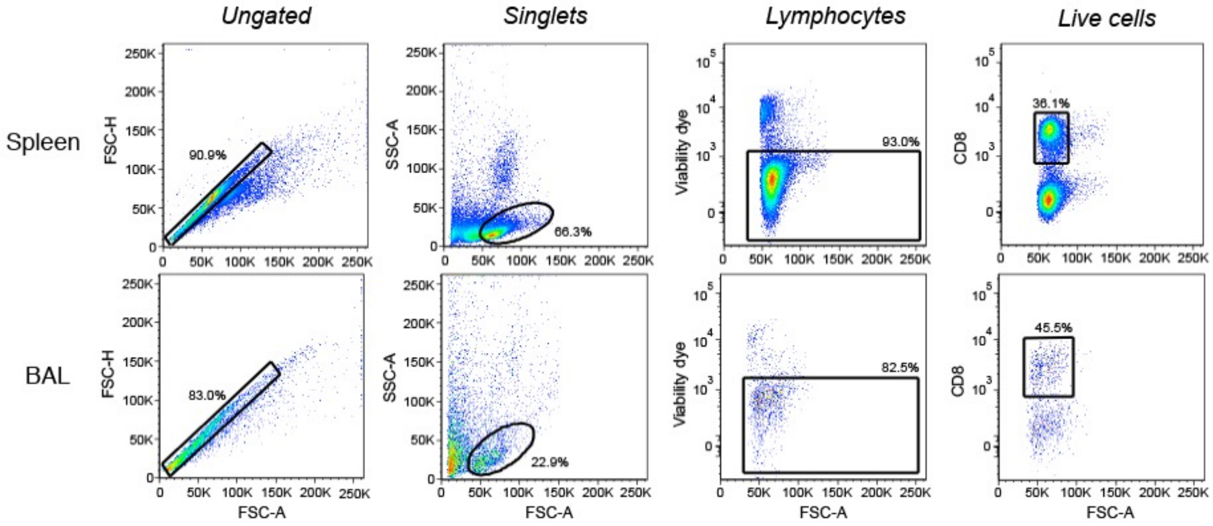

Supplementary Fig. 7. Gating strategy for CD8$^+$ T cells stained for intracellular cytokine production. Naïve B6 mice were infected i.n. with 1000 pfu PR8 IAV, and spleen and bronchoalveolar lavage (BAL) harvested 10 days later. Splenocytes and BAL cells were enriched for CD8$^+$ T cells, stimulated for 5 hours in the presence or absence of 1 uM individual peptides and stained for cytokine analysis. Shown is the gating strategy for the identification of single cells, lymphocytes, live cells, and CD8$^+$ cells prior to analysis of cytokine staining in splenocytes (upper panel, relates to plots shown in Fig. 5) and BAL cells (lower panel, relates to plots shown in Supplementary Figure 6).

Supplementary Tables

Supplementary Table 1. MRM transitions for isotopic IAV peptides

Peptide	Sequence*	Q1 m/z (charge)	Q3 m/z (ion)	Optimal CE (eV)
$NP_{366-374}$	ASNE**N**METM	516.7007 (+2)	522.2124 (b_5)	15.7
			653.2528 (b_6)	15.7
			782.2954 (b_7)	15.7
			883.3431 (b_8)	15.7
NP_{36-43}	IGRFY**I**QM	517.7819 (+2)	443.2564 (b_7^{+2})	21.8
			637.3457 (b_5)	33.8
			757.4469 (b_6)	24.8
			885.5055 (b_7)	21.8
			921.4724 (y_7)	27.8
NP_{55-63}	R**L**IQNSLTI	532.8304 (+2)	632.3952 (b_5)	34.3
			719.4272 (b_6)	31.3
			804.5164 (a_7)	34.3
			832.5113 (b_7)	22.3
$PA_{224-233}$	SS**L**ENFRAYV	596.8072 (+2)	508.2878 (y_4)	25.6
			655.3562 (y_5)	31.6
			769.3991 (y_6)	31.6
			834.4104 (b_7)	25.6
			898.4417 (y_7)	31.6
$PB1F2_{62-70}$	LSLRNPI**L**V	516.3379 (+2)	457.7684 (b_8^{+2})	18.7
			584.3515 (b_5)	36.7
			831.5524 (y_7)	24.7
			914.5895 (b_8)	24.7
$PB1_{703-711}$	SSYRRP**V**GI	347.5342 (+3)	426.7396 (b_7^{+2})	18.4
			441.2528 (a_8^{+2})	24.4
			455.2503 (b_8^{+2})	18.4
			477.2816 (y_8^{+2})	18.4

$PB1_{653-660}$	K**N**MEYDAV	488.2247 (+2)	509.2357 (b_4)	23.5
			672.2991 (b_5)	20.5
			787.3260 (b_6)	20.5
			858.3631 (b_7)	17.5
$PB2_{227-234}$	VYIE**V**LHL	496.2964 (+2)	487.3271 (y_4)	25.0
			616.3697 (y_5)	23.7
			729.4538 (y_6)	20.7
			892.5171 (y_7)	22.0
$NS2_{114-121}$	RTFSFQ**L**I	509.7933 (+2)	492.2565 (b_4)	36.5
			639.3249 (b_5)	27.5
			767.3835 (b_6)	21.5
			859.4898 (a_7)	30.5
			887.4847 (b_7)	18.5
$NS2_{8-16}$	SFQDI**L**LRM	565.311 (+2)	419.2435 (y_3)	28.0
			532.3276 (y_4)	26.7
			652.4288 (y_5)	35.7
			767.4557 (y_6)	29.7
			895.5143 (y_7)	28.0
$M1_{128-135}$	MG**L**IYNRM	502.7601 (+2)	309.1705 (b_3)	24.0
			420.2024 (y_3)	24.0
			583.2657 (y_4)	24.0
			696.3498 (y_5)	25.5
			816.4510 (y_6)	24.0
			873.4724 (y_7)	27.0
$M1_{227-237}$	AGLKND**L**LENL	603.8437 (+2)	538.2964 (b_{10}^{+2})	22.4
			719.4160 (b_7)	31.4
			832.5000 (b_8)	28.4
			961.5426 (b_9)	25.4
$M1_{207-216}$	SQARQM**V**QAM	578.2857 (+2)	571.2947 (b_5)	42.3
			702.3352 (b_6)	36.3

			807.4174 (b_7)	27.3
			935.4760 (b_8)	25.5
			940.4735 (y_8)	26.0
HA$_{402-409}$	MN**I**QFTAV	465.7450 (+2)	494.2505 (b_4)	13.3
			641.3189 (b_5)	13.3
			742.3666 (b_6)	13.3
			813.4037 (b_7)	13.3
HA$_{304-311}$	SS**L**PYQNI	461.2374 (+2)	634.3195 (y_5)	18.0
			683.3472 (b_6)	16.3
			797.3902 (b_7)	16.3
HA$_{41-49}$	VT**V**THSVNL	488.2787 (+2)	670.3519 (y_6)	23.4
			730.4126 (b_7)	20.4
			775.4341 (y_7)	23.4
			844.4556 (b_8)	21.0
			876.4818 (y_8)	24.0
HA$_{308-316}$	YQNIHP**V**TI	545.7998 (+2)	656.3151 (b_5)	26.0
			799.4705 (y_7)	26.0
			858.4501 (b_7)	23.0
HA$_{389-399}$	NGITNK**V**NTVI	589.8422 (+2)	285.1557 (b_3)	27.8
			733.4235 (b_7)	33.8
			847.4665 (b_8)	27.8
			894.5287 (y_8)	24.8
			948.5141 (b_9)	26.0
NA$_{181-190}$	SGPDNGA**V**AV	446.7238 (+2)	294.2056 (y_3)	15.5
			599.2420 (b_7)	23.0
			704.3242 (b_8)	15.5
			775.3613 (b_9)	15.5
NA$_{181-191}$	SGPDNGA**V**AVL	503.2658 (+2)	599.2420 (b_7)	30.0
			704.3242 (b_8)	24.0
			775.3613 (b_9)	21.0

			874.4297 (b_{10})	15.0

*Isotopic heavy amino acids are denoted in underlined bold type.

Supplementary Table 2. MRM transitions for identified IAV peptides.

Peptide	Sequence	Q1 m/z (charge)	Q3 m/z (ion)	Optimal CE (eV)
$NP_{366-374}$	ASNENMETM	513.6970 (+2)	516.2049 (b_5)	15.7
			647.2454 (b_6)	15.7
			776.2879 (b_7)	15.7
			877.3356 (b_8)	15.7
NP_{36-43}	IGRFYIQM	514.2733 (+2)	439.7478 (b_7^{+2})	21.8
			637.3457 (b_5)	33.8
			750.4297 (b_6)	24.8
			878.4883 (b_7)	21.8
			914.4553 (y_7)	27.8
NP_{55-63}	RLIQNSLTI	529.3218 (+2)	625.3780 (b_5)	34.3
			712.4100 (b_6)	31.3
			797.4992 (a_7)	34.3
			825.4941 (b_7)	22.3
$PA_{224-233}$	SSLENFRAYV	593.2986 (+2)	508.2878 (y_4)	25.6
			655.3562 (y_5)	31.6
			769.3991 (y_6)	31.6
			834.4104 (b_7)	25.6
			898.4417 (y_7)	31.6
$PB1F2_{62-70}$	LSLRNPILV	512.8293 (+2)	4542898 (b_8^{+2})	18.7
			584.3515 (b_5)	36.7
			794.4883 (b_7)	24.7
			824.5352 (y_7)	24.7
			907.5724 (b_8)	24.7
$PB1F2_{62-72}$	LSLRNPILVFL	642.9055 (+2)	383.7503 (a_7^{+2})	39.4
			454.2898 (b_8^{+2})	30.4
			794.4833 (b_7)	33.4
			907.5724 (b_8)	39.4

$PB1_{703-711}$	SSYRRPVGI	345.5296 (+3)	423.7327 (b_7^{+2})	18.4
			438.2459 (a_8^{+2})	24.4
			452.2434 (b_8^{+2})	18.4
			474.2747 (y_8^{+2})	18.4
$PB1_{653-660}$	KNMEYDAV	485.2209 (+2)	503.2282 (b_4)	23.5
			666.2916 (b_5)	20.5
			781.3185 (b_6)	20.5
			852.3556 (b_7)	17.5
$PB2_{227-234}$	VYIEVLHL	493.2895 (+2)	481.3133 (y_4)	25.0
			610.3559 (y_5)	23.7
			723.4400 (y_6)	20.7
			886.5033 (y_7)	22.0
$NS2_{114-121}$	RTFSFQLI	506.2847 (+2)	492.2565 (b_4)	36.5
			639.3249 (b_5)	27.5
			767.3835 (b_6)	21.5
			852.4726 (a_7)	30.5
			880.4676 (b_7)	18.5
$NS2_{8-16}$	SFQDILLRM	561.8024 (+2)	419.2435 (y_3)	28.0
			532.3276 (y_4)	26.7
			645.4116 (y_5)	35.7
			760.4386 (y_6)	29.7
			888.4971 (y_7)	28.0
$M1_{128-135}$	MGLIYNRM	499.2515 (+2)	302.1533 (b_3)	24.0
			420.2024 (y_3)	24.0
			583.2657 (y_4)	24.0
			696.3498 (y_5)	25.5
			809.4338 (y_6)	24.0
			866.4553 (y_7)	27.0
$M1_{227-237}$	AGLKNDLLENL	600.3352 (+2)	534.7878 (b_{10}^{+2})	22.4
			712.3988 (b_7)	31.4

			825.4829 (b_8)	28.4
			954.5255 (b_9)	25.4
M1$_{207-216}$	SQARQMVQAM	575.2788 (+2)	571.2947 (b_5)	42.3
			702.3352 (b_6)	36.3
			801.4036 (b_7)	27.3
			929.4622 (b_8)	25.5
			934.4597 (y_8)	26.0
HA$_{402-409}$	MNIQFTAV	462.2364 (+2)	487.2333 (b_4)	13.3
			634.3017 (b_5)	13.3
			735.3494 (b_6)	13.3
			806.3865 (b_7)	13.3
HA$_{304-311}$	SSLPYQNI	461.2374 (+2)	634.3195 (y_5)	18.0
			676.3301 (b_6)	16.3
			790.3730 (b_7)	16.3
HA$_{41-49}$	VTVTHSVNL	485.2718 (+2)	670.3519 (y_6)	23.4
			724.3988 (b_7)	20.4
			769.4203 (y_7)	23.4
			838.4417 (b_8)	21.0
			870.4680 (y_8)	24.0
HA$_{308-316}$	YQNIHPVTI	542.7929 (+2)	656.3151 (b_5)	26.0
			793.4567 (y_7)	26.0
			852.4363 (b_7)	23.0
HA$_{389-399}$	NGITNKVNTVI	586.8353 (+2)	285.1557 (b_3)	27.8
			727.4097 (b_7)	33.8
			841.4526 (b_8)	27.8
			888.5149 (y_8)	24.8
			942.5003 (b_9)	26.0
HA$_{467-476}$	SQLKNNAKEI	382.5546 (+3)	389.2395 (y_3)	21.9
			460.2766 (y_4)	18.9
			688.3624 (y_6)	24.9

$NA_{181-190}$	SGPDNGAVAV	443.7169 (+2)	288.1918 (y_3)	15.5
			599.2420 (b_7)	23.0
			698.3104 (b_8)	15.5
			769.3475 (b_9)	15.5
$NA_{181-191}$	SGPDNGAVAVL	500.2589 (+2)	599.2420 (b_7)	30.0
			698.3104 (b_8)	24.0
			769.3475 (b_9)	21.0
			868.4159 (b_{10})	15.0

Supplementary Table 3. Summary of detection limit and linear range for each isotopic peptide.

Peptide	Sequence*	Allele	Linear range (fmol)	R	LLOQ (fmol)
NP_{36-43}	IGRFY**I**QM	K^b	0.1 to 500	0.9879	0.1
NP_{55-63}	R**L**IQNSLTI	D^b	0.1 to 500	0.9941	0.1
$PB1_{653-660}$	K**N**MEYDAV	K^b	0.1 to 1000	0.9766	0.1
$PB2_{227-234}$	VYIE**V**LHL	K^b	0.1 to 500	0.9690	0.1
$NS2_{8-16}$	SFQDI**L**LRM	K^b	0.1 to 500	0.9888	0.1
$M1_{128-135}$	MG**L**IYNRM	K^b	0.1 to 500	0.9568	0.1
$M1_{227-237}$	AGLKND**L**LENL	D^b	0.1 to 500	0.9819	0.1
$M1_{207-216}$	SQARQM**V**QAM	D^b	0.1 to 500	0.9834	0.1
$HA_{402-409}$	MN**I**QFTAV	K^b	0.1 to 500	0.9318	0.1
$HA_{304-311}$	SS**L**PYQNI	K^b	0.1 to 500	0.9863	0.1
HA_{41-49}	VT**V**THSVNL	D^b& K^b	0.1 to 500	0.8774	0.1
$HA_{308-316}$	YQNIHP**V**TI	D^b	0.1 to 1000	0.9285	0.1
$HA_{389-399}$	NGITNK**V**NTVI	D^b	0.1 to 500	0.9789	0.1
$NA_{181-190}$	SGPDNGA**V**AV	D^b	0.1 to 500	0.9829	0.01
$NA_{181-191}$	SGPDNGA**V**AVL	D^b	0.1 to 500	0.9975	0.01

*Isotopic heavy amino acids are denoted in underlined bold type.
