# Supplementary material for: Quantification of epitope abundance reveals the effect of direct and cross-presentation on influenza CTL responses
Source: Nat Commun. 2019 Jun 28;10:2846. doi: 10.1038/s41467-019-10661-8 (PMC6599079; doi:10.1038/s41467-019-10661-8)
Supplement: Supplementary file 3 — Description of Additional Supplementary Files [file 41467_2019_10661_MOESM3_ESM.docx]

**Description of Supplementary Files**

**File Name:** Supplementary Data 1

**Description:** Protein Pilot data for all identified peptides.

**File Name:** Supplementary Data 2

**Description:** Quantitation and kinetics of peptide presentation following direct and cross-presentation.
