# Supplementary material for: Quantification of epitope abundance reveals the effect of direct and cross-presentation on influenza CTL responses
Source: Nat Commun. 2019 Jun 28;10:2846. doi: 10.1038/s41467-019-10661-8 (PMC6599079; doi:10.1038/s41467-019-10661-8)
Supplement: Supplementary file 6 — Reporting Summary [file 41467_2019_10661_MOESM6_ESM.pdf]

nature research

Corresponding author(s): Nathan Croft
Anthony W Purcell
Nicole L La Gruta

Last updated by author(s): May 16, 2019

Reporting Summary

Nature Research wishes to improve the reproducibility of the work that we publish. This form provides structure for consistency and transparency in reporting. For further information on Nature Research policies, see Authors & Referees and the Editorial Policy Checklist.

Statistics

For all statistical analyses, confirm that the following items are present in the figure legend, table legend, main text, or Methods section.

n/a	Confirmed	
☐	☒	The exact sample size (*n*) for each experimental group/condition, given as a discrete number and unit of measurement
☐	☒	A statement on whether measurements were taken from distinct samples or whether the same sample was measured repeatedly
☐	☒	The statistical test(s) used AND whether they are one- or two-sided<br>*Only common tests should be described solely by name; describe more complex techniques in the Methods section.*
☐	☒	A description of all covariates tested
☐	☒	A description of any assumptions or corrections, such as tests of normality and adjustment for multiple comparisons
☐	☒	A full description of the statistical parameters including central tendency (e.g. means) or other basic estimates (e.g. regression coefficient) AND variation (e.g. standard deviation) or associated estimates of uncertainty (e.g. confidence intervals)
☐	☒	For null hypothesis testing, the test statistic (e.g. *F*, *t*, *r*) with confidence intervals, effect sizes, degrees of freedom and *P* value noted<br>*Give P values as exact values whenever suitable.*
☐	☒	For Bayesian analysis, information on the choice of priors and Markov chain Monte Carlo settings
☐	☒	For hierarchical and complex designs, identification of the appropriate level for tests and full reporting of outcomes
☐	☒	Estimates of effect sizes (e.g. Cohen's *d*, Pearson's *r*), indicating how they were calculated

Our web collection on statistics for biologists contains articles on many of the points above.

Software and code

Policy information about availability of computer code

Data collection	Analyst (SCIEX) versions TF 1.7.1 (for TripleTOF 5600+) & 1.6.3 (for QTRAP 5500).
Data analysis	PeakView (SCIEX) version 2.2. Graphpad Prism 7. Skyline 64-bit (v4.1.0.18169). Microsoft Excel (2016).

For manuscripts utilizing custom algorithms or software that are central to the research but not yet described in published literature, software must be made available to editors/reviewers. We strongly encourage code deposition in a community repository (e.g. GitHub). See the Nature Research guidelines for submitting code & software for further information.

Data

Policy information about availability of data

All manuscripts must include a data availability statement. This statement should provide the following information, where applicable:

- Accession codes, unique identifiers, or web links for publicly available datasets
- A list of figures that have associated raw data
- A description of any restrictions on data availability

MS datasets have been uploaded to the following online repository: http://www.peptideatlas.org/PASS/PASS01317 (password: MB5448fp)

Field-specific reporting

Please select the one below that is the best fit for your research. If you are not sure, read the appropriate sections before making your selection.

☒ Life sciences ☐ Behavioural & social sciences ☐ Ecological, evolutionary & environmental sciences

For a reference copy of the document with all sections, see nature.com/documents/nr-reporting-summary-flat.pdf

Life sciences study design

All studies must disclose on these points even when the disclosure is negative.

Sample size	Sample size was determined to allow the application of appropriate statistical tests as defined within the text.
Data exclusions	No data were excluded from the study
Replication	All experimental findings were reproducible as evidenced by the fact that similar results were obtained when experiments were repeated
Randomization	This is not relevant to this study as there was no allocation of individual samples or animals into experimental groups.
Blinding	Blinding was not relevant for the reason described above

Reporting for specific materials, systems and methods

We require information from authors about some types of materials, experimental systems and methods used in many studies. Here, indicate whether each material, system or method listed is relevant to your study. If you are not sure if a list item applies to your research, read the appropriate section before selecting a response.

Materials & experimental systems

n/a	Involved in the study	
☐	☒	Antibodies
☐	☒	Eukaryotic cell lines
☒	☐	Palaeontology
☐	☒	Animals and other organisms
☒	☐	Human research participants
☒	☐	Clinical data

Methods

n/a	Involved in the study	
☒	☐	ChIP-seq
☐	☒	Flow cytometry
☒	☐	MRI-based neuroimaging

Antibodies

Antibodies used

Anti-mouse CD8-PacBlue (53-6.7), BD Pharmingen - 558106
Anti-influenza A NP - FITC monoclonal antibody (D67J), eBioscience - MA1-7322
Anti-mouse IFN-FITC (XMG1.2), BD Pharmingen - 554411
Anti-mouse TNF-APC (MP6-XT22), BD Pharmingen - 506308
Anti-mouse H-2Db-FITC (28-14-8), eBioscience - 11-5999-82
Anti-mouse H-2Kb-FITC (AF6-88.5.5.3), eBioscience - 11-5958-82
Anti-mouse H-2Kb+SIINFEKL (25-D1.16), eBioscience - 14-5743-81
Anti-mouse IgG1-PE (X56), BD Biosciences - 340270
Anti-mouse H-2Kb monoclonal Ab (Y-3), in house purified from hybridoma ATCC line HB-176
Anti-mouse H-2Db monoclonal Ab (28-14-8S), in house purified from hybridoma ATCC line HB-27

Validation

All commercial antibodies were vaildated as part of the QC process, as well as being validated in the lab on mouse cells. In house antibodies were validated on mouse cells known to express targets of interest.

Eukaryotic cell lines

Policy information about cell lines

Cell line source(s)

DC2.4, Dr Kenneth Rock, University of Massachusetts, Worcester, MA
LET1 cells - Dr Paul Thomas, St Jude Children's Research Hospital, Memphis Tn, USA
A549 cells - in house
Mutu DC cell line - Dr Hans Acha-Orbea, University of Lausanne, Epalinges, Switzerland
EL-4 cells - in house
EG7 - in house

Authentication

All cell lines were authenticated by observing cellular morphology (LET1), expression of particular markers such as mouse class I (DC2.4, Mutu DC, EL4), expression of Kb+SIINFEKL (EG7), an absence of mouse MHCI (A549) or by cross-presentation capacity (Mutu DC).

Mycoplasma contamination

Cell lines either tested negative for mycoplasma or were not tested for mycoplasma.

Commonly misidentified lines
(See ICLAC register)

no commonly misidentified cell lines were used

Animals and other organisms

Policy information about studies involving animals; ARRIVE guidelines recommended for reporting animal research

Laboratory animals	C57Bl/6 (B6, H-2b) WT mice and OT-1 transgenic mice expressing a TCR specific for the OVA257– 264 SIINFEKL peptide in complex with H2-Kb were used
Wild animals	No wild animals were used
Field-collected samples	This study did not involve samples collected from the field
Ethics oversight	All the experimental protocols involving animals were approved by Animal Experimentation Ethics Committees at the University of Melbourne or at Monash University.

Note that full information on the approval of the study protocol must also be provided in the manuscript.

Flow Cytometry

Plots

Confirm that:

☒ The axis labels state the marker and fluorochrome used (e.g. CD4-FITC).

☒ The axis scales are clearly visible. Include numbers along axes only for bottom left plot of group (a 'group' is an analysis of identical markers).

☒ All plots are contour plots with outliers or pseudocolor plots.

☒ A numerical value for number of cells or percentage (with statistics) is provided.

Methodology

Sample preparation	Cells were processed either from in vitro cultures or from spleen or bronchoalveolar lavage of influenza A infected mice. Cells from tissue were processed into single cell suspensions by pressing through a sieve and were enriched for CD8 cells by incubating on plastic for 1h at 37 degrees.
Instrument	A BD LSRII or Fortessa X20 (LLC) was used for analysis
Software	FlowJo software (FlowJo LLC, Ashland) was used for analysis, FACSDIVA software (BD Biosciences) was used for acquisition
Cell population abundance	Although sorted cell populations were not used directly in this study, post-sort analysis routinely involves a purity check by running a post-sort sample back through analyser. Purity is always >97%.
Gating strategy	The CD8+ T cells analysed in this study were first gated for live cells using a LIVE/DEAD Fixable AquaBlue or Near InfraRed Viability Dye, lymphocytes using FSC/SSC characteristics, and CD8+ cells were clearly distinct from CD8- cells after staining. Cells producing cytokine were identified by including a 'no peptide' control, gates were set using this negative control, and the % cells deemed positive occurred following subtraction of the % found in this negative control. Isotype controls were used to set gates for other experiments (e.g. Supp Fig 3), and EL4 cells were used as a negative control for positive 25D1.16 staining of EG7 (Supp Fig. 5).

☒ Tick this box to confirm that a figure exemplifying the gating strategy is provided in the Supplementary Information.
