# Supplementary figures and images for: Quantification of epitope abundance reveals the effect of direct and cross-presentation on influenza CTL responses
Source: Nat Commun. 2019 Jun 28;10:2846. doi: 10.1038/s41467-019-10661-8 (PMC6599079; doi:10.1038/s41467-019-10661-8)

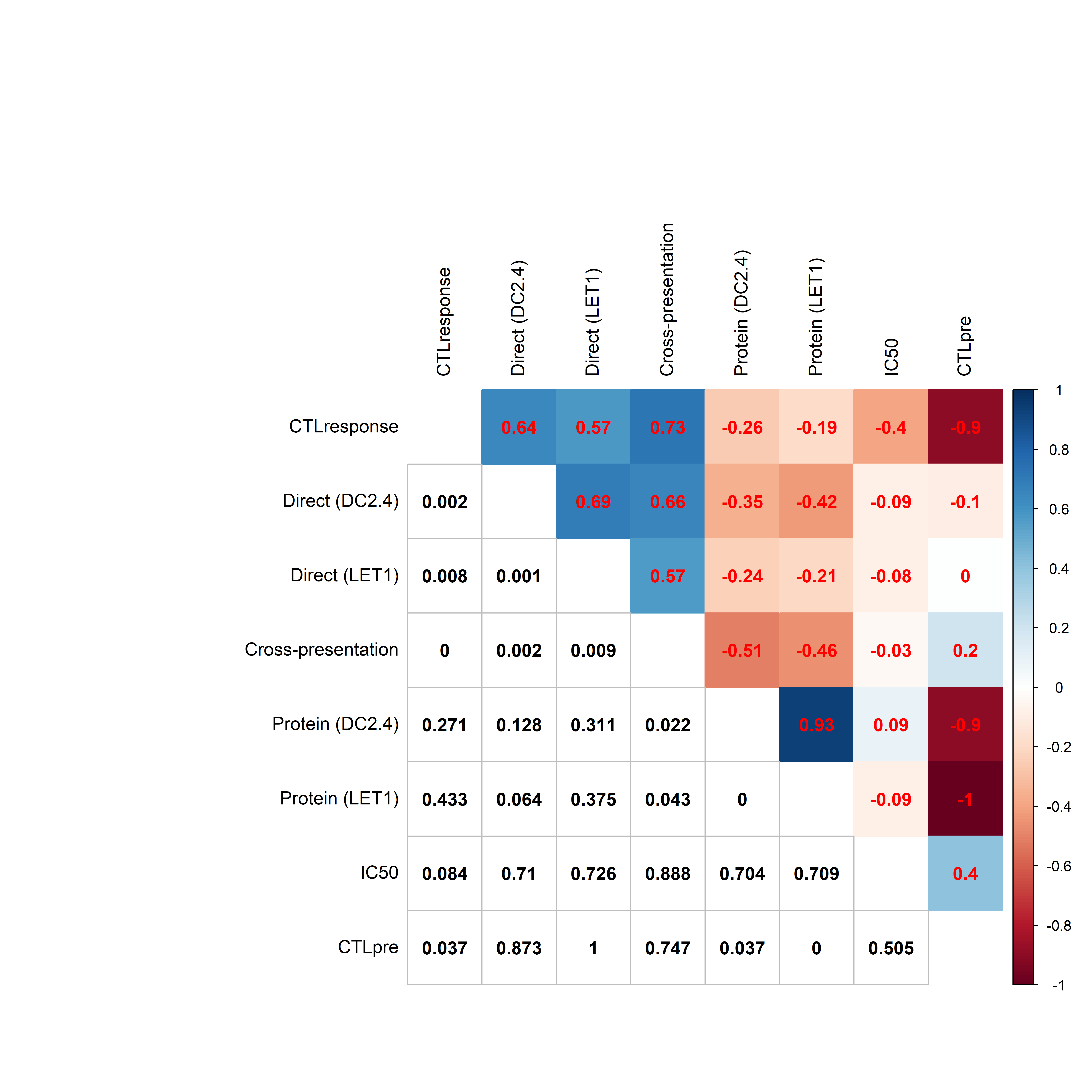

Supplement: Supplementary file 7 — Supplementary Software 1 [file 41467_2019_10661_MOESM7_ESM.zip › Analysis Code/results/corrplot.png]

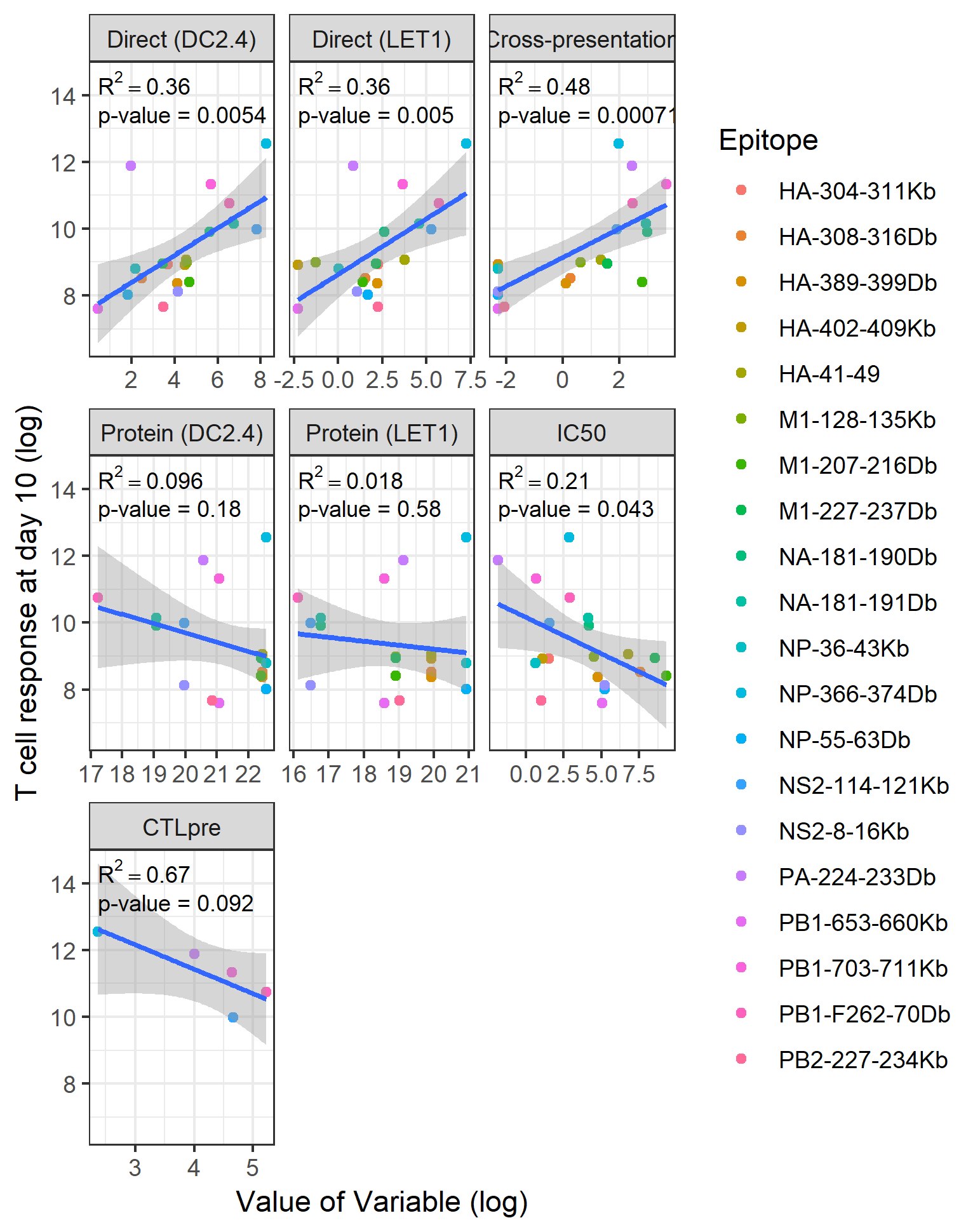

Supplement: Supplementary file 7 — Supplementary Software 1 [file 41467_2019_10661_MOESM7_ESM.zip › Analysis Code/results/uni-lm-plot.png]
